# Building capacity for estimating fire emissions from tropical peatlands; a worked example from Indonesia

**DOI:** 10.1038/s41598-023-40894-z

**Published:** 2023-09-01

**Authors:** Haruni Krisnawati, Liubov Volkova, Budiharto Budiharto, Franky Zamzani, Wahyu C. Adinugroho, Muhammad A. Qirom, Christopher J. Weston

**Affiliations:** 1 Research Center for Ecology and Ethnobiology, Research Organization for Life Sciences and Environment, National Research and Innovation Agency (BRIN), Jl. Raya Jakarta-Bogor KM. 46, Cibinong, Bogor, 16911 Indonesia; 2 Ministry of Environment and Forestry, Jl. Gatot Subroto, Jakarta, 10270 Indonesia; 3 Faculty of Science, School of Agriculture, Food and Ecosystem Sciences, The University of Melbourne, Creswick, VIC 3363 Australia; 4 Directorate of Greenhouse Gas Inventory and Monitoring Reporting and Verification, Directorate General of Climate Change, Ministry of Environment and Forestry, Jl. Gatot Subroto, Jakarta, 10270 Indonesia; 5 Directorate of Climate Change Mitigation, Directorate General of Climate Change, Ministry of Environment and Forestry, Jl. Gatot Subroto, Jakarta, 10270 Indonesia; 6 National Research and Innovation Agency (BRIN), Banjarbaru, 70721 Indonesia

**Keywords:** Ecosystem services, Tropical ecology, Wetlands ecology, Fire ecology

## Abstract

Tropical peatlands are globally significant in the terrestrial carbon cycle as they are comprised of a large forest carbon sink and a large peat carbon store—both of which can potentially be exchanged with the atmosphere on decadal time frames. Greenhouse gas emissions from fire-disturbance and development of tropical peatlands over the last few decades, and the potential for ongoing emissions, highlights the need for policy to slow or halt emissions and to activate mechanisms to sequester carbon through restoration of degraded peatlands. The UN REDD + scheme provides a means for developing countries to receive payments for avoided deforestation and forest degradation, but the steps to achieve REDD+ compliance are rigorous and the details required can be a barrier to activating benefits—especially for peatlands where repeated cycles of fire interrupt forest recovery and create a range of recovery classes. Therefore, to improve estimates of peat fire emissions and of carbon balance of tropical peatlands, the biomass and combustion factor parameters need to be developed and applied according to forest recovery stage. In this study we use published activity data from the extensive 1997 fires in the peatlands of Indonesian Borneo to detail a transparent and accountable way to estimate and report emissions from tropical peatland fires. This example for estimating and reporting emissions is provided to assist REDD+ countries to efficiently develop their capacity for improving emissions estimates from fire-impacted tropical peatlands.

## Introduction

Greenhouse gas (GHG) emissions arising from the fire disturbance of tropical peatlands have been highlighted in several recent studies^1^ because of the potential for ongoing emissions and also for opportunities to sequester carbon (C) through restoration of degraded peatlands^2^. Tropical peatlands are a globally significant terrestrial store of C estimated at around 105 Pg^2^. The approximately 13.43 million ha of tropical peatlands in Indonesia^3^, accounts for about 50% of the global tropical peatland carbon store^4^.

Forest biomass burning is a major source of global anthropogenic emissions, with CO_2_ emissions from wildfires averaging 2.2 Pg C yr^−1^^5^—equivalent to 22% of the global fossil fuel emissions^6^. Reported emissions from the 1997 Indonesian peat fires range from 810 to 2570 Tg C (0.81–2.57 Gt C) using field based estimates^7^, to the 500 Tg C estimated by the Global Fire Emissions Database (GFEDv4) model^5^. The field-based estimates were derived using a simple multiplication of, (1) peat burn depth derived from the ground measurements, and (2) peat carbon content, (3) aboveground biomass—both derived from the literature—and where a single value was applied to both degraded peatlands and to intact forests, and (4) the area burnt derived using remote sensing approach (details below). The GFEDv4 estimates were based on a complex biogeochemical model (Carnegie–Ames–Stanford Approach, CASA) that was adjusted for revised fuel consumption parameters using field based observations and burnt areas derived from satellite observations.

Reducing atmospheric CO_2_ concentration and keeping global warming at bay was the focus of the recent COP 26 Climate Summit in Glasgow 2021, and reducing smoke and emissions specifically from peat fires has gained national and international significance as a mechanism for addressing climate change. For these reasons the Food and Agriculture Organisation (FAO) of the United Nations (UN) has recently declared that improving the assessment of GHG emissions from peatlands is a global strategic priority^8^. Improving the assessment of GHG emissions from peatlands will allow for the development of policy that establishes incentives to achieve peatland restoration at a country scale, particularly through schemes such as REDD + . Several studies over the last two decades have documented the rapid degradation of tropical peatlands, with clearing and drainage often followed by frequent fires and development of agriculture and plantations over large areas of former peat swamp forest^9, 10^. The opportunity for conserving and restoring tropical peatlands is a priority for climate change mitigation as well as biodiversity conservation^11^ and water resource protection.

**Figure 1:**
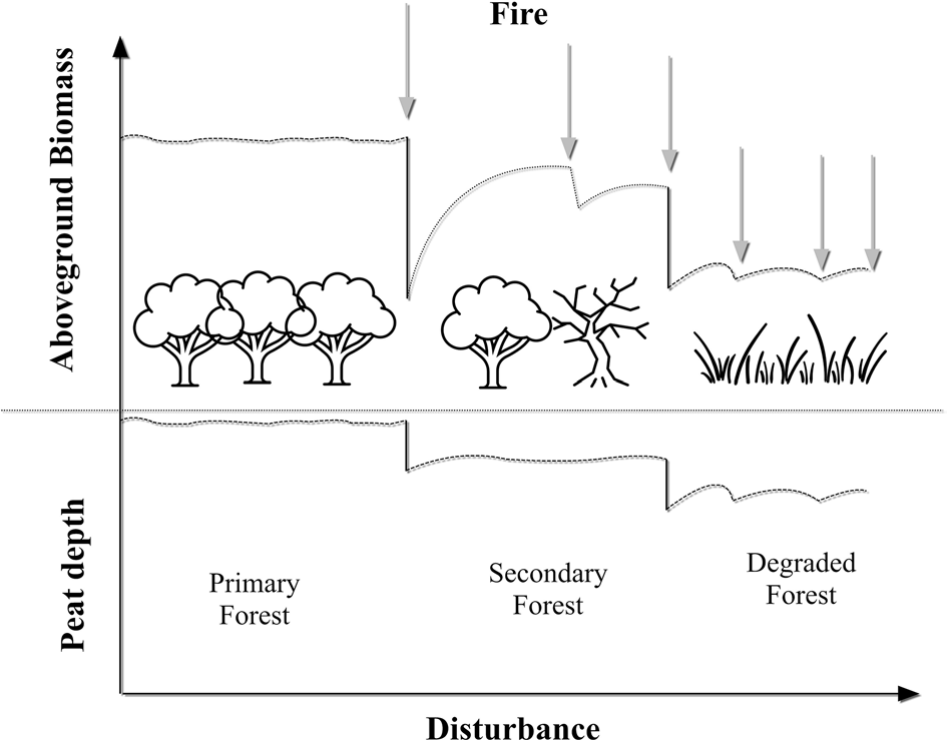
A schematic diagram of the relationship between fire frequencies and peat swamp forests Aboveground Biomass and peat depth.

International pressure on Indonesia to reduce emissions from peat fires is ongoing, yet the magnitude of emissions *released* to the atmosphere is uncertain. This uncertainty is partly due to different approaches to account for emissions from the diversity of peatland landscapes that have evolved from the initial fire occurrence, that may or may not have followed initial logging. Peat swamp forests (PSF) degraded by logging and fire subsequently become either secondary forests, or plantations on peat, or repeatedly fire-impacted degraded peatlands. Intact or primary peat swamp forests store more carbon aboveground and in the peat layer than burnt and recovering forests or repeatedly burnt and degraded forest (Fig. 1), where the latter are generally dominated by shrubs and grasses and have a shallower peat layer than less disturbed forests^12^.

Recognizing the varying outcomes of either recovery or continued degradation of peatland landscapes following disturbance is important for carbon and emission accounting, as forest condition sets the starting point for selecting the most appropriate parameters for emissions calculations. However most published studies rely on the default parameters provided in the Guideline of the Intergovernmental Panel on Climate Change IPCC^13^ that apply to primary forests, and rarely adopt parameters that are more appropriate to the burning of already degraded peat swamp forests. Moreover, as we mentioned above, highly cited publications on the amount of emissions released from peat fires in Indonesia have applied simplified, non-IPCC methods for estimating emissions, and have also not distinguished between primary and already degraded peatlands in making their emissions estimates^7, 14^.

Initial methodologies for estimating emissions from biomass burning were published by the IPCC in 1996^15^. The parameters required for estimating emissions are the activity data (AD, or area burnt) and emission factors (EF)—Eq. (1).

1 $$E=Area\cdot EF$$

The EF is estimated as the mass of the fuel available for combustion (*M*), multiplied by the fuel combustion factor (*Cf*) and a gas specific emission factor (*G*_*i*_). *Cf* refers to how much fuel is combusted (or released to the atmosphere) and *G*_*i*_ refers to the amount of *i* greenhouse gas (i.e., CO, CH_4_, N_2_O) emitted per kg of fuel burnt—Eq. (2).

2 $$EF=M \cdot {C}_{f} \cdot {G}_{i}$$

In the case of peatlands, separate EFs are required for aboveground and peat layers—as both are combusted in fires (Fig. 1), thus information on mass of peat (M_PEAT_) and AGB and their corresponding *Cf* and *Gi* will be required for the estimates.

Mass of peat (${M}_{PEAT}$) combusted in fires is estimated as peat bulk density (BD) multiplied by the depth of burnt peat (*h*)—Eq. (3).

3 $${M}_{PEAT} =BD\cdot h$$

Emissions are reported in CO_2_-equivalent and global warming potential (GWP) for each of the reported gases (CH_4_, N_2_O, CO) is also required.

Thus, to estimate emissions for *i* gas from peat, we need:

4 $${E}_{i\,PEAT}=Area \, burnt\cdot (BD\cdot h \cdot {Cf}_{PEAT} \cdot {G}_{i\,PEAT} \cdot {GWP}_{i})$$

We also need to estimate emissions for *i* gas from Above Ground Biomass (AGB):

5 $${E}_{i\, AGB}=Area \, burnt\cdot (AGB \cdot {Cf}_{AGB} \cdot {G}_{i\, ABG} \cdot {GWP}_{i})$$

Thus, the total emissions will be the sum of emissions from peat and AGB for all reported *i* gases (e.g., CO_2_, CO, N_2_O, CH_4_)—Eq. (6):

6 $$E={(E}_{CO2\, AGB}+ {E}_{CO2\, PEAT})+\left({E}_{C{O\, AGB}}+ {E}_{C{O\, PEAT}}\right)+{(E}_{N2O\, AGB}+ {E}_{N2O\, PEAT})+\left({E}_{CH{4\, AGB}}+ {E}_{CH{4\, PEAT}}\right)$$

As we mentioned above, using the same M for primary or intact forests and for degraded peatlands will either over or underestimate emissions (Fig. 1). Therefore, emissions estimates should be stratified by peatland cover classes (or fire frequencies)—Eq. (7).

7 $${E}_{Total}={E}_{primary\, forest,\, 1 fire }+ {E}_{secondary\, forest,\, 2-3\, fires }+ {E}_{degraded\, forest,\, >3\, fires}$$

As research advances and as technologies progress revision of Eq. (4) and (5) parameters i.e., AGB, BD, h, Gi, Cf, acts to improve emissions estimates and reduce uncertainties. One good example of continuous updates in the emission equation parameters is for peat combustion factor (*Cf*) of Eq. (4). The widely used IPCC 2014 Supplement^13^ states on page 2.39: “For all organic soil fires, the default combustion factor is 1.0, since the assumption is that all fuel is combusted (Yokelson et al., 1997)”. Thus a single study is used for the peat related emissions estimates worldwide. However, on a detailed examination of the study referred too^16^, there is no mention of a *Cf* . Thus a great number of peat emissions estimates in reports and publications around the world are based on a default *Cf* that is not supported by the literature. The IPCC has been alerted to this oversight and is considering an update to default *parameters* for calculating emissions from peat fires. The worked example presented here applies peat combustion factors (*Cf*), that have been stratified by fire frequency, according to the authors recent peer-reviewed study^17^.

For biomass burning, the United Nations Framework Convention on Climate Change (UNFCCC) encourage non-Annex I Parties to provide information on anthropogenic emissions by source for CO_2_, CH_4_, N_2_O (Decision 17/CP.8, annex, paragraph14), CO, NO_x_, and non-methane VOCs (Decision 17/CP.8, annex, paragraph16). Following from this requirement, a further challenge for developing countries is that many of the parameters required for emission estimates are derived from scientific publications that are only available at considerable cost via journal subscriptions or paywall access to the required articles. Also, parameters for some compounds are not available, such as GWP for NOx^18^. For many gases, such as non-methane volatile organic compounds (NMVOC) or NOx—an abbreviation for nitric oxide (NO) and nitrogen dioxide (NO_2_), specific *G*_*i*_ are not easy to identify for staff responsible for the national level reporting, who may not have a chemistry background. Thus, on the one hand, it is a *good practice* to report emissions as comprehensively as possible, but on the other hand many countries lack the experience and capacity to do so, or elect not to report all gases.

The UNFCCC established the REDD+ scheme as a means for developing countries to receive result-based payments for reducing emissions from deforestation and forest degradation. To achieve REDD+ compliance each country is required to establish a forest reference level (FRL) or forest reference emissions level (FREL) and a process for monitoring, verification and reporting of annual forest carbon stock changes and emissions. The uptake and overall success of the REDD+ scheme requires that each country develops expertise in GHG emissions reporting—a significant step given the complexities of the policy, the underlying science, and the many steps to implementation. For these reasons capacity building is an essential component of climate change mitigation and adaptation initiatives that are promoted through UNFCCC mechanisms. Countries where emissions from biomass burning is one of the major sources of national emissions need to have appropriately skilled and experienced staff, thus training and capacity building is essential. Emissions reporting also relies on relevant peer-reviewed publications to provide a transparent and defensible scientific basis for parameters selected and applied in emissions calculations.

As an example of the complex nature of emissions reporting, the Government of Indonesia (GoI) did not report peat fire emissions for its first FREL submitted in 2016, due to a high level of uncertainty in the parameters used in the estimates, including those as the Appendix^19^. Emissions from peat fires were included in their second FREL submission in 2022 due to improved capacities and due to newly published parameters for peat combustion factors (*Cf *_*PEAT*_)^17^. This is a significant development for the improvement of global emissions estimates from peatlands.

In this study we provide a simple example of estimating emissions from peat fires using the IPCC methodology in a transparent and accountable way. We stratified our peat swamp forests into three Peatland cover classes: (1) Primary or secondary long-unburnt peat swamp forests, (1st fire); (2) secondary peat swamp forests burnt in 2–3 consecutive fires (2nd–3rd fires), and (3) degraded peatlands, frequently burnt, shrub-dominated (> 3 fires). We combined data from various sources (details below) to separate emissions by fire frequencies. While our intention was to report emissions for all major GHGs as encouraged by the UNFCCC, a review of the literature for *G*_*i*_ and GWP revealed that we can estimate emissions for only four major GHGs (CO_2_, CO, CH_4_ and N_2_O).

## Methods

### Study rationale and approach

**Figure 2:**
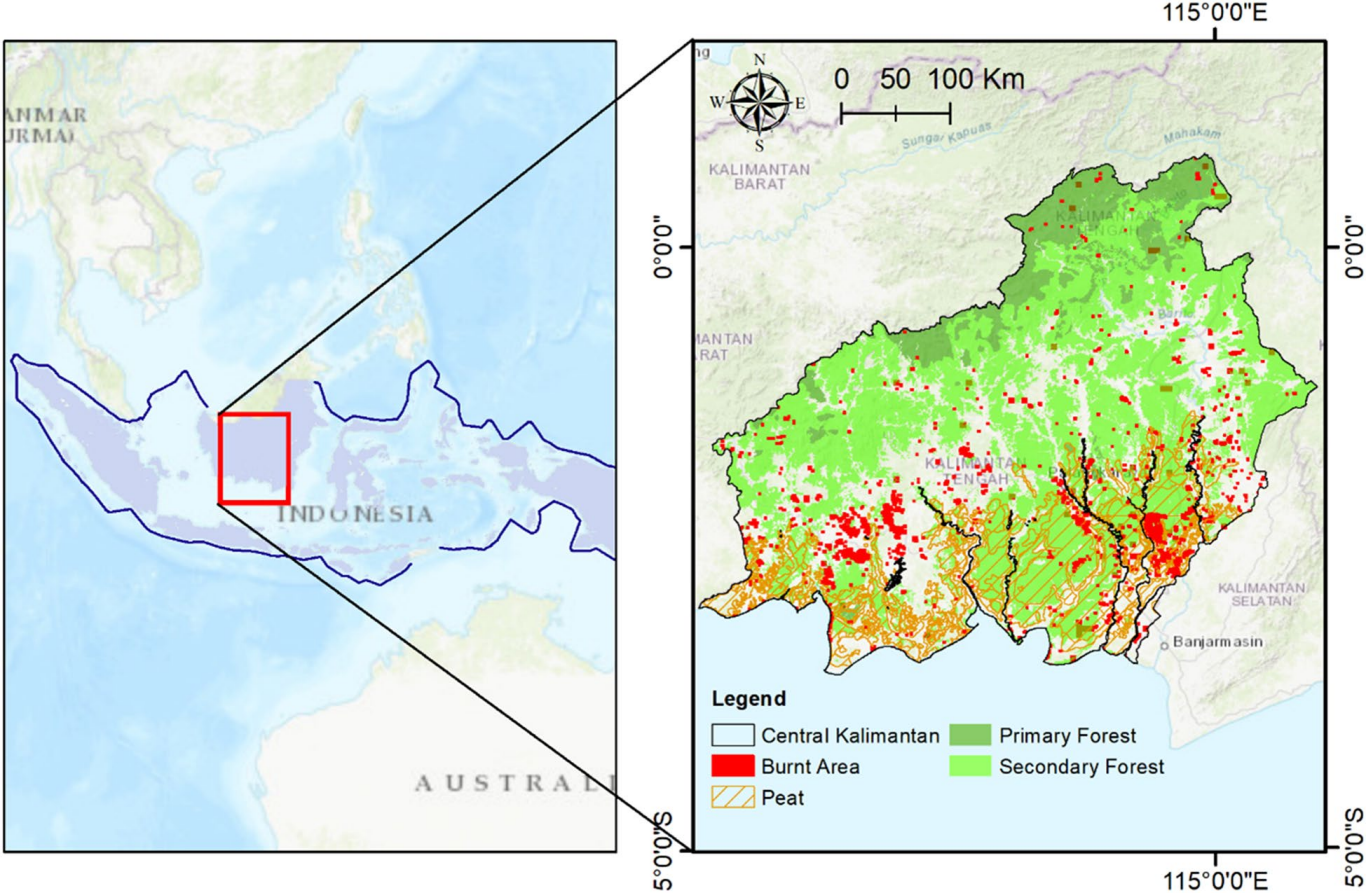
Area of peat swamp forests burnt in 1997 fires. Map created using the Free and Open Source QGIS.

To describe a worked example for the calculation of GHG emissions from major landscape fires in tropical peatlands we used published activity data (i.e. area burnt by peat landcover class) from the 1997 fires in peatlands of Central Kalimantan, Indonesia^7^. The 1997 fires were unprecedented in the history of Indonesia, burning through 729,500 million hectares of peatlands (Fig. 2). We also chose the 1997 fires because of their wide coverage in the scientific literature, including fire emissions estimates equivalent to between 13 and 40% of the mean annual global carbon emissions accounted from fossil fuels^7^.

The methodology applied in this study estimated emissions separately for peat, and for the aboveground biomass, using the emissions equations (Eq. 3–7) as described above (a Tier 2 IPCC method).

### Datasets

AGB and peat bulk density parameters were derived from three datasets based on field measurements in peat swamp forests of Kalimantan^17, 20, 21^ according to the categories of fire frequency as described above. The bulk density and biomass parameters were bootstrapped for 10,000 iterations and the average and 95% CI values were used in the calculations.

The other parameters such as h, *Gi*, *Cf* were derived from the literature (Table 1).

**Table 1:** Parameters required to estimate emissions from peat and AGB and their sources.

Aboveground biomass, AGB			Peat				To estimate CO_2_-eq
*AGB*	${Cf}_{AGB}$	${G}_{i\_AGB}$	$h$	$BD$	${Cf}_{PEAT}$	${G}_{i\_PEAT}$	*GWP*
This study dataset	^17^	^22^	^23, 14^	This study dataset	^17^	^24^	^18^

### Estimating mass and EF for peat and AGB

As described above in Eq. (3), peat mass (M_PEAT_) was estimated from peat bulk density (BD) and the depth (*h)* of peat burnt, while peat EF was estimated from peat mass (M_PEAT_) and a combustion factor (C*f*_PEAT_) according to fire frequency (Eq. 4) and (Table 2).

**Table 2:** Parameters required to estimate M_PEAT_ and EF_PEAT_ and their sources.

Fire frequency	N	$BD$, g cm^−3^	$h$, cm	Ref for h	M_PEAT_, Mg ha^−1^	*Cf*_PEAT_	Ref for Cf_PEAT_	EF_PEAT,_Mg ha^−1^
					(BD*h*100)			(M_PEAT_ *Cf_PEAT_)
1st fire	110	0.169 (0.156, 0.182)	33	^14^	556 (513; 598)	0.54	^17^	300 (277; 323)
2nd –3rd fire	36	0.205 (0.189, 0.221)	10	^23^	205 (189; 221)	0.195	^17^	40.0 (36.8; 43.1)
> 3 fires			2	^23^	41.03 (37.8; 44.2)	0.195	^17^	8.0 (6.7;9.2)

Values are means (95%CI).

AGB was estimated as the sum of biomass components—trees, litter, deadwood and pyrogenic carbon, pending data availability (Table 3).

**Table 3:** Parameters required to estimate M_ABG_ and EF_AGB_ and their sources.

Fire frequency	Number of observations	$AGB$, Mg ha^−1^	Cf_AGB_	Ref for Cf _AGB_	EF_AGB,_ Mg ha^−1^
1st fire	104	276 (256; 295)	0.564	^17^	155 (144; 166)
2nd –3rd fire	61	46.6 (29.3; 63.9)	0.564	^17^	26.3 (16.5; 36.1)
> 3 fires		negligible			

Values are means (95%CI).

### Developing fire area map

A fire area map for the 1997 fires in Kalimantan was developed using QGIS 3.3 (Open Source Geospatial Foundation) by overlaying the NOAA-AVHRR Hotspot 1998 layer with the Mawas Burnt area 1997, the Natural Forest Cover 1996, and the peat distribution and administration boundary for Kalimantan. The 1996 Natural Forest Cover map was provided by the Ministry of Environment and Forestry, and the Peat Distribution Map was provided by the Ministry of Agriculture. The Mawas Burnt Area for 1997 was extracted from^25^.

## Results and discussion

### GHG emissions from peat layer and aboveground biomass

Emissions were higher from the 1st fire compared to more frequent fire categories, reflecting the greater depth of peat burnt in first fire (Table 4). Total emissions decreased with increased fire frequency as the depth of peat burnt decreased.

**Table 4:** Emission Factors and GHG Emissions from peat layer.

Fire frequency	EF_PEAT_, Mg ha^−1^	Emissions from peat layer Mg CO_2_ eq ha^−1^				
		CO_2_	CO	CH_4_	N_2_O	Total Emission
	EF_PEAT_* (*Table 2*)*	E_i_ = EF_PEAT_ * G_i_ * GWP_i_ * 10^−3^				*∑E*_*i*_
1st fire	300 (277; 323)	469 (433; 505)	166 (153;178)	71.4 (65.9,76.8)	17.9 (16.5;19.3)	725 (669;780)
2nd–3rd fire	40.0 (36.8; 43.1)	62.6 (57.6; 67.5)	22.1 (20.4; 23.8)	9.51 (8.76; 10.3)	2.38 (2.19; 2.57)	96.6 (89.0; 104)
> 3 fires	8.0 (6.7;9.2)	25.6 (21.5;29.57)	9.05 (7.6;10.5)	3.89 (3.27;4.49)	0.97 (0.62;1.12)	39.5 (33.2;45.6)

G_i_ = Gas specific emission factor: CO_2_ = 1564 g kg^−1^; CO = 291 g kg^−1^; N_2_O = 0.2 g kg^−1^; CH_4_ = 9.51 g kg^−1^.

GWP: CO_2_ = 1; CO = 1.9; CH_4_ = 25; N_2_O = 298.

Emissions from AGB followed the same pattern as for peat layer with the highest emissions from long unburnt forests (Table 5).

**Table 5:** Emission Factors and GHG Emissions for Aboveground biomass.

Fire frequency	EF_AGB_, Mg ha^−1^	Emissions Mg CO_2_ eq ha^−1^				
		CO_2_	CO	CH_4_	N_2_O	Total Emission
	EF_AGB_ (see Table 3)	E_i_ = EF_AGB_ * G_i_ * GWP_i_ * 10^−3^				*∑E*_*i*_
1st fire	155 (144; 166)	252 (234; 270)	30.7 (28.6; 32.9)	25.3 (23.5; 27.1)	9.28 (8.63; 9.93)	317 (295; 340)
2nd–3rd fire	26.3 (16.5; 36.1)	42.6 (26.7; 58.4)	5.19 (3.26; 7.12)	4.27 (2.68; 5.85)	1.56 (0.98; 2.14)	53.6 (33.6; 73.6)

Gas specific emission factors (*G*_*i*_): CO_2_ = 1620 g kg^−1^; CO = 104 g kg^−1^; N_2_O = 0.2 g kg^−1^; CH_4_ = 6.5 g kg^−1^.

GWP: CO_2_ = 1; CO = 1.9; CH_4_ = 25; N_2_O = 298.

### Carbon released from 1997 peat fires—comparison of estimates reported in Page et al. 2002 with the estimates from this study

As mentioned in the introduction, the total burnt area for Kalimantan from the study of^7^ was applied to each of the peatland cover class reported (Table 6), and emission equation parameters derived in this study were applied to estimate total emissions for the peat layer (Table 3) and for AGB (Table 4).

**Table 6:** Comparison of the estimates of carbon released from 1997 peat fires using the emission data from Tables 4 and 5 and as reported in^7^.

Peatland cover class	Total study area, 10^3^ ha	Damage in class, %	Area burnt, ha	Total emissions per cover class, t CO_2_ eq ha^−1^	Total emissionsGt CO_2_ eq	C loss, Gt
			*A*	*E* = *from Tables (4* + *5)*	*E*_*TOTAL*_ = A*E*10^−9^	*C*_*LOSS*_ = *E*_*TOTAL*_ * 12/44
Primary PSF	217.1	4.5	9,769.5	1,042.0	0.01	0.0028
Logged over PSF	1,070.70	29.2	312,644.4	725.0	0.23	0.0618
Fragmented PSF	79	70	55,300.0	778.6	0.04	0.0117
Riverine swamp forest	16.8	7.1	1,192.8	1,042.0	0.001	0.0003
Fragmented riverine swamp forest	130.5	15.7	20,488.5	778.6	0.02	0.0044
Mangrove forest	88.4	14.6	12,906.4	1,042.0	0.01	0.0037
Forest mosaics	257.7	54.1	139,415.7	778.6	0.11	0.0296
Bushland	207.9	45.2	93,970.8	737.4	0.07	0.0189
Swampy grasslands	75.8	41	31,078.0	737.4	0.02	0.0063
Total peatland	2,143.90	33.9	726,782.1		0.51	0.14
Page et al. 2002 study	2,143.90	33.9	726,782.1			0.24–0.28

Total study area and damage in class are derived from Tables 1 and 2 of^7^.

Our emissions results for the study area are 0.14 Gt C, half the reported 0.24–0.28 Gt C in^7^. The 50% lower emissions estimate can be attributed to the following factors: (1) changes in *Cf*_*PEAT*_: the^7^ study used *Cf*_*PEAT*_ = 1, assuming that 100% of peat up to 50 cm depth was emitted to the atmosphere over the whole of burnt peatlands, which is likely an overestimation and an oversimplification of the heterogeneity of burnt peatland; (2) depth of peat burnt: 50 cm of peat burnt was used in^7^—later studies^14, 23^ proposed a shallower depth of peat burnt—33 cm—the value used in our estimates; (3) AGB: the study by^7^ used a single AGB carbon density of 250 t C ha^−1^ (i.e. 500 t ha^−1^)—applied to primary PSF, logged over PSF and bushlands alike. We note that AGB of 500 t ha^−1^ reflects highly productive forests and our dataset, which included data from multiple sources with 110 data points had only two forests with AGB reported above 500 t ha^−1^. Surely, logged over PSF and bushlands would not have 500 t ha^−1^ stored in AGB (Fig. 1).

The average emission estimates in our study of 1.92 × 10^−7^ Gt C ha^−1^ (i.e., the total emission of 0.14 Gt C divided by the total area burnt of 726,782.1 ha), demonstrates that the 1997 fires released between 0.47 Gt C, an intermediate estimate (1.92 × 10^−7^ Gt C ha^−1^ × 2,441,000 ha) and an upper estimate 1.31 Gt C (1.92 × 10^−7^ Gt C ha^−1^ × 6,804,668 ha), where 2,441,000 ha and 6,804,668 ha are the peat area burnt over the entire Indonesia extracted from^7^. This is a half of the 0.81–2.57 Gt C emissions previously reported. Our estimates match well with the top–down estimates of GFEDv4 of 0.5 Gt C released from the 1997 fires. While GFEDv4 estimates produced a reasonable assessment of Indonesian 1997 peat fires emissions, it may be technically challenging for some countries to use GFEDv4. The example emissions calculations shown in this study are relatively simple, can be completed in an excel spreadsheet, and also do not require an in depth knowledge of rather complex and lengthy IPCC methodologies.

To better enable the development of climate change mitigation actions we believe that building the capacity of countries that aspire to REDD+ payments, through international collaborations with countries more experienced in emission reporting, is a priority for the UNFCCC COP meetings to address. This study can help to guide communities from REDD+  countries, and anyone interested more generally in emissions reporting, to make informed IPCC-method compliant estimates.

## Conclusions

In this study we demonstrate the application of IPCC compliant methodology to estimate and report GHG emissions (CO_2_, CO, CH_4_, N_2_O) from tropical peatland fires impacting both aboveground and peat carbon pools. The method is transparent and accounts for a range of fire regimes observed in former peat swamp forest areas over recent decades—to account for either different stages of forest recovery or ongoing chronic fire-disturbance cycles. This nuancing of GHG reporting is especially important in degraded PSF areas because of their potential contribution to either carbon storage (sequestration), or to GHG emissions. This example is provided to assist countries aspiring to participate in the REDD+ scheme to more efficiently navigate the compliance requirements for estimating and reporting emissions from tropical peatlands.

The recently published peat combustion factor data^17^ has been used in Indonesia’s 2nd FREL submitted to the UNFCCC in early 2022, and it has been assessed by UNFCCC Technical experts. In addition, Indonesia’s national GHG inventory reporting is also being updated following the use of the new data in the 2nd FREL report.

We suggest that a more detailed calculation of peat fire emissions according to fire frequency class is achievable in future reporting. It will require detailed activity data to differentiate the areas that have been burnt once, twice, or multiple times which would require resources and high technology/capacity to run the process at the national scale.

We hope that more mechanisms will be developed to increase capacity in developing countries for REDD+ compliance so that they can lead climate change mitigation actions at local to regional and to global levels.

## Data Availability

The datasets used and/or analysed during the current study available from the corresponding author on reasonable request.
